# 1677. Trends in Antibiotic Resistance Among Uropathogens in the Pediatric Population: A Multicenter Experience in the US

**DOI:** 10.1093/ofid/ofad500.1510

**Published:** 2023-11-27

**Authors:** Leena B Mithal, David Chu, Catherine Forster, Ibukun Kalu, John Wiener, Shan Sun, Joseph Fishbein, Mehreen Arshad

**Affiliations:** Lurie Children's/Northwestern, Chicago, Illinois; Lurie Children's/Northwestern, Chicago, Illinois; University of Pittsburg Medical Center, Pittsburgh, Pennsylvania; Duke University, Durham, NC; Duke Children's, Durham, North Carolina; Ann and Robert H. Lurie Children's Hospital, Chicago, Illinois; Lurie Children's/Northwestern, Chicago, Illinois; Northwestern University/Lurie Children's Hospital of Chicago, Chicago, IL

## Abstract

**Background:**

Urinary tract infections (UTIs) are common infections in children. Overuse of antibiotics has led to increasing antibiotic resistance among uropathogens in adults; however, data on pediatric trends have not been previously reported. Our objective was to characterize antibiotic resistance trends in uropathogens among children at three tertiary care hospitals across the US.

**Methods:**

Microbiologic data from positive urine cultures ( >20,000 CFU/ml) in children 0 – 18 yrs of age between 1/1/2010 and 12/31/2020 were obtained from electronic medical records. Yearly antibiotic agent-specific resistance rates were calculated based on culture, patient, and organism level data.

**Results:**

A total of 17,747 positive individual urine cultures were analyzed. *E. coli* was the most common organism isolated. Across all three institutions, rates of resistance against ampicillin/sulbactam (average rate 24%, IQR 3.21%) and trimethoprim/sulfamethoxazole (average rate 30%, IQR 6.42%) among *E. coli* remained high but stable over the study period. However, resistance against levofloxacin as well as third and fourth generation cephalosporins generally increased over this period across all institutions (Figure 1) and age groups (Figure 2). Combined antibiograms of all three institutions for 2019 and 2020 showed high rates of resistance against ampicillin/sulbactam and trimethoprim/sulfamethoxazole among Enterobacterales in general and variable resistance against ceftriaxone.

**Figure:**
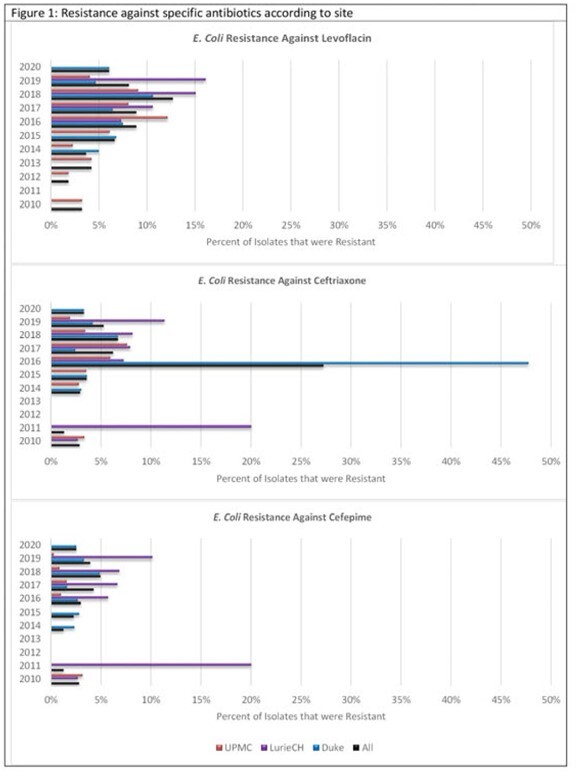

**Figure:**
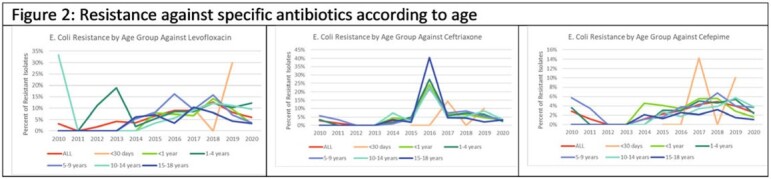

**Conclusion:**

Over the last 10 years, there have been rising rates of resistance to broad spectrum antibiotics, including beta-lactams and quinolones, among uropathogens collected from a pediatric cohort with an increasing trend starting in 2015-2016. While we were not able to distinguish patients with community acquired UTI from hospital acquired UTIs, we noted with interest that the prevalence of resistant uropathogens even among the youngest age groups (< 30 days and < 1 year) trends along with the older children. This suggests a likely community reservoir of multi-drug resistant gram-negative bacteria. Colonization by resistant uropathogens has implications for empiric antibiotic choice, limited oral therapy options, and clinical outcomes which necessitate further study.

**Disclosures:**

**Ibukun Kalu, MD**, Pfizer: Institutional support for clinical trial

